# Transmission Dynamics of Monkeypox Virus in Nigeria during the Current COVID-19 Pandemic and Estimation of Effective Reproduction Number

**DOI:** 10.3390/vaccines10122153

**Published:** 2022-12-15

**Authors:** Salihu Sabiu Musa, Zainab Umar Abdullahi, Shi Zhao, Umar Muhammad Bello, Nafiu Hussaini, Abdulrazaq Garba Habib, Daihai He

**Affiliations:** 1 Department of Applied Mathematics, Hong Kong Polytechnic University, Hong Kong SAR, China; 2 Operational Research Center in Healthcare, Near East University TRNC, Mersin 10, Nicosia 99138, Turkey; 3 Department of Mathematics, Kano University of Science and Technology, Wudil, Kano 713101, Nigeria; 4 Department of Biological Sciences, Federal University Dutsinma, Katsina 821101, Nigeria; 5 JC School of Public Health and Primary Care, Chinese University of Hong Kong, Hong Kong SAR, China; 6 Department of Physiotherapy and Paramedicine, School of Health and Life Sciences, Glasgow Caledonian University, Glasgow G4 0BA, UK; 7 Department of Mathematical Sciences, Bayero University Kano, Kano 700241, Nigeria; 8 Department of Medicine, Bayero University Kano, Kano 700241, Nigeria

**Keywords:** monkeypox virus, COVID-19, effective reproduction number, epidemic, public health

## Abstract

Monkeypox virus (MPXV) continues to pose severe threats to global public health, especially in non-endemic areas. Like all other regions, Africa faces potential public health crises due to the ongoing COVID-19 pandemic and other infectious disease outbreaks (such as Lassa fever and malaria) that have devastated the region and overwhelmed the healthcare systems. Owing to the recent surge in the MPXV and other infections, the COVID-19-control efforts could deteriorate and further worsen. This study discusses the potential emergencies of MPXV transmission during the current COVID-19 pandemic. We hypothesize some of the underlying drivers that possibly resulted in an increase in rodent-to-human interaction, such as the COVID-19 pandemic’s impact and other human behavioral or environmental factors. Furthermore, we estimate the MPXV time-varying effective reproduction number ($R_{t}$) based on case notification in Nigeria. We find that $R_{t}$ reached a peak in 2022 with a mean of 1.924 (95% CrI: 1.455, 2.485) and a median of 1.921 (95% CrI: 1.450, 2.482). We argue that the real-time monitoring of $R_{t}$ is practical and can give public health authorities crucial data for circumstantial awareness and strategy recalibration. We also emphasize the need to improve awareness programs and the provision of adequate health care resources to suppress the outbreaks. These could also help to increase the reporting rate and, in turn, prevent large community transmission of the MPXV in Nigeria and beyond.

## 1. Background

Monkeypox is a viral disease caused by the monkeypox virus (MPXV) [1]. The MPXV belongs to the *Orthopoxvirus* genus under the family of *Poxviridae* and was first discovered in 1958 [2]. The first human case was recorded in the Democratic Republic of Congo (DRC) in 1970 [2]. Since then, the virus has spread substantially due to the rapid surge in human urbanization and environmental deterioration [3]. The recent emergence of MPXV in non-endemic countries, especially in Europe, America, and some Asian countries, poses a great concern to public health globally. This compelled the World Health Organization (WHO) to declare the ailment a Public Health Emergency of International Concern (PHEIC) in its meeting held on 21 July 2022 [2]. This is the second time in two years (and the seventh since 2007) that the WHO has declared a global emergency of a disease outbreak [2]. Like other regions, COVID-19 hit Africa badly, causing millions of cases of morbidity and mortality since the beginning of the outbreak in China in December 2019 [4]. Thus, investigating the syndemic effects of the pandemic and other infectious diseases, such as the MPXV, is imperative.

From 1 January to 20 September 2022, the virus affected more than 63 thousand individuals worldwide, in over 106 locations; over 99% were reported from 99 non-endemic countries/locations that historically have not reported MPXV cases [5,6]. Surprisingly, more than 95% of the currently reported MPXV cases have been linked to males, with an excessive number of cases identifying themselves as gay, bisexual, and men who have sex with men (MSM) [2]. This is perhaps one of the most devastating scenarios as there was no documented prior evidence of the sexual transmission of the virus [7]. However, the known transmission route of the virus encompassed human-to-human contact, including sexual contact [7,8]. In addition, occupational transmission has been reported [9], and it should be regarded in the light of public health uncertainty.

Monkeypox, a zoonotic viral infection, primarily occurs in the tropical rainforest regions of Central and West Africa and is occasionally exported to non-endemic countries, such as America and those in Europe and Asia. The MPXV is transmitted to humans via contact with an infected person (including sexual contact), animal, or contaminated material. It is worth noting that the main transmission route for MPXV in Nigeria is through zoonotic as well as person-to–person transmission. The symptoms may include fever, headache, swollen lymph nodes, which could result in serious medical complications, lymphadenopathy, and a rash that forms blisters and then crusts all over the body [10]. The incubation period ranges between 5 and 21 days. The estimated mean serial incubation interval is about 9.8 (95% CI: 5.9–21.4) days [2,11]. The serial interval is considered the period between an infected person’s symptoms and those of the subsequently infected person [12].

Following the first human cases of the MPXV discovered in the DRC in 1970, the Nigerian health authorities followed and monitored the situation very closely and increased surveillance to prevent transmission. Fortunately, no MPXV case was reported until the subsequent year, i.e., in 1971, when two human MPXV cases were reported [13]. The first case was that of a four-year-old girl from the present Abia state of the southern part of Nigeria who developed symptoms of the MPXV (including fever and skin rash) on 9 April 1971. The mother was subsequently infected following contact tracing [14]. Then, a few confirmed/probable cases were reported between 1971 and 1978; however, no mortality was recorded. After nearly four decades (i.e., in early 2017), the human transmission of the MPXV reemerged in Nigeria with higher transmissibility and severity. By the end of 2017, Nigeria recorded about 115 confirmed cases of MPXV [15]. The first MPXV cases out of Africa happened in September 2018, when infected individuals travelled out of Nigeria to the United Kingdom [16].

Understanding the clinical differential diagnosis of the MPXV requires the analysis of other skin-related infections in different settings, such as chickenpox, measles, syphilis, and bacterial skin infections. For instance, lymphadenopathy during the prodromal stage of illness can be considered a clinical feature to differentiate the MPXV from smallpox or chickenpox [2]. The treatment of the MPXV infection focuses primarily on reducing symptoms, managing complications, and preventing long-term sequelae. However, since the MPXV and the smallpox viruses are genetically similar, antiviral medications and vaccines developed to treat infected people and shield susceptible individuals from smallpox can also be used to treat and protect against the MPXV infection [2,5]. Tecovirimat (TPOXX) is an effective antiviral medication for use against MPXV infection, especially for patients with weak immune systems who are more likely to develop severe disease, even though most MPXV patients can recover fully within 2–4 weeks without requiring medical treatment [5]. The global eradication of smallpox in the 1970s and 1980s led to the termination of its vaccination, which provided immunity against the MPXV infection [1]. Thus, most people under 50 years of age have no immunity against the MPXV, making them more vulnerable to the virus.

The MPXV is a virus with high transmissibility [17]. It is endemic in many Central and Western African countries but rarely seen outside these regions [2]. The recent emergence in non-endemic countries, which has been linked to an outbreak in the United Kingdom [18], has caused serious public health concerns, leading to the declaration of the virus as the PHEIC [2]. Consequently, sporadic outbreaks of the virus followed, affecting more than 70 countries worldwide [6]. Early model predictions estimated the basic reproduction number (Ro, i.e., the average number of secondary MPXV cases produced by a single case during the whole infectious period in a community without immunity or any intervention) of the MPXV to be > 1 in MSM populations and < 1 in other settings [2]. For instance, Du et al. [11] estimated the *Ro(t)* of the MPXV for the multicounty outbreak as 1.29 (95% CrI: 1.26, 1.33) by aggregating all the cases in 70 countries as of 22 July 2022.

In this study, we aim to investigate the epidemiological scenario of the MPXV in Nigeria and the potential threat it might have posed, especially during the current COVID-19 pandemic. We also estimate $R_{t}$ using the serial interval approach (serial interval [SI] is the time lag between the symptoms onset of a primary case and its secondary cases) [12,19]. We use the MPXV data from January 2017 to August 2022 to quantify the instantaneous transmissibility of the outbreak.

## 2. Material and Methods

### 2.1. Data

We obtained the number of laboratory confirmations of the MPXV cases data for January 2017 through to August 2022 from the situation reports released by the Nigeria Centre for Disease Control [20]. We obtained the COVID-19 situation reports from the WHO COVID-19 surveillance dashboard system for the data from January 2020 to July 2022 [4]. The time-series analysis for the MPXV and COVID-19 was performed using the R statistical software (version 4.2.0; The R Foundation for Statistical Computing, Vienna, Austria); see https://www.r-project.org/ accessed on 6 September 2022. Nigeria is a significant trading partner with many countries, including the United States, China, Germany, and France. As a result, it experiences frequent business travel, which increases the susceptibility to disease infections and helps to overwhelm its healthcare system, particularly during pandemic periods [21]. Therefore, understanding the current epidemiological scenarios of the MPXV outbreaks in Nigeria, and considering the current COVID-19 pandemic, is essential to the overall management and prevention plans for the two diseases in Nigeria and elsewhere.

### 2.2. Effective Reproduction Number Estimation Analysis

The time-varying effective reproduction number, $R_{t}$, was used to measure the MPXV outbreak’s instantaneous transmissibility [22,23]. Using the $R_{t}$ estimation approach described by [22,24], the time-varying reproduction number, $R_{t}$, at time *t*, is given by

(1) $$R_{t}=\frac{I\left(t\right)}{\int_{0}^{\infty }\omega \left(s\right)I\left(t-s\right)ds}$$

where $I\left(t\right)$ denotes the MPXV incidence rate at time t. The convolution expression $\int_{0}^{\infty }\omega_{s}I\left(t-s\right)ds$ in the above equation represents the measurement of the total infectiousness at time t. The term $\omega \left(s\right)$ denotes the MPXV serial interval (SI) distribution which describes how the infectiousness was distributed when the illness was active. In the current research, we employed the three different SIs for the MPXV estimated by Guo et al. [25] and Ward et al. [10] to quantify the $R_{t}$ and compare the results. Guo et al. [25] used the right truncation and the sampling bias-adjusted techniques to estimate the SI mean and standard deviation as 5.6 and 1.5 days, respectively. Ward et al. [10] used two different models to estimate the SI. Those are: (1) the interval censoring corrected (ICC) model, by which the SI mean and standard deviation were estimated at 8 and 9 days, respectively, and (2) the interval censoring right truncation corrected (ICRTC) model, by which the SI mean and standard deviation were estimated at 9.5 and 10.9 days, respectively. Thus, by employing the above estimates in Equation (1), we obtained the $R_{t}$ as shown in Figure 2a–f, and the results are summarized in Table 1 and Table 2. Note that $R_{t}$ = *R_eff_.*

## 3. Results and Discussions

### 3.1. Current Epidemiological Scenarios of MPXV and COVID-19 in Nigeria

The recent surge of the MPXV during the COVID-19 pandemic in endemic and non-endemic countries is problematic to the already fragile healthcare systems of many countries, including Nigeria. COVID-19 has become one of the most continuously evolving pandemics, leading to serious problems for humanity in the past few years [4].

**Figure 1:**
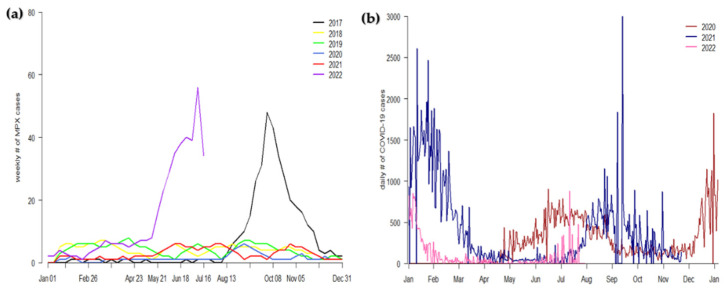
Panel (**a**): time-series simulation results of the monkeypox virus in Nigeria from 2017 to 2022. Panel (**b**): time series simulations result of the COVID-19 epidemic situation in Nigeria from 2020 to 2022. The cases data were obtained from [4,20].

In Figure 1a, we presented the time-series simulation results of the MPXV scenarios for the data from January 2017 to July 2022 to illustrate the epidemic curve of the virus that has mainly increased in 2022 in Nigeria. This is a rare situation that has not occurred since 2017. The data show that high MPXV infections were observed in 2017 and 2022, reaching an all-time peak in 2022. This demonstrates that though the COVID-19 pandemic (which started early in 2020) did not immediately affect the transmission of the MPXV infection in 2020 and 2021, it could be one of the reasons for the current increase in MPXV transmission. However, more data are needed to support this assertion. To respond to the situation and enhance MPXV control and prevention in Nigeria, all suspected, probable, and confirmed cases are usually isolated to prevent further community transmission. In Figure 1b, we presented the time-series simulations results of the COVID-19 pandemic scenarios in Nigeria for the data from January 2020 to July 2022 in order to illustrate the current epidemiological situations of the COVID-19 pandemic in Nigeria.

### 3.2. Effective Reproduction Number Estimation

**Figure 2:**
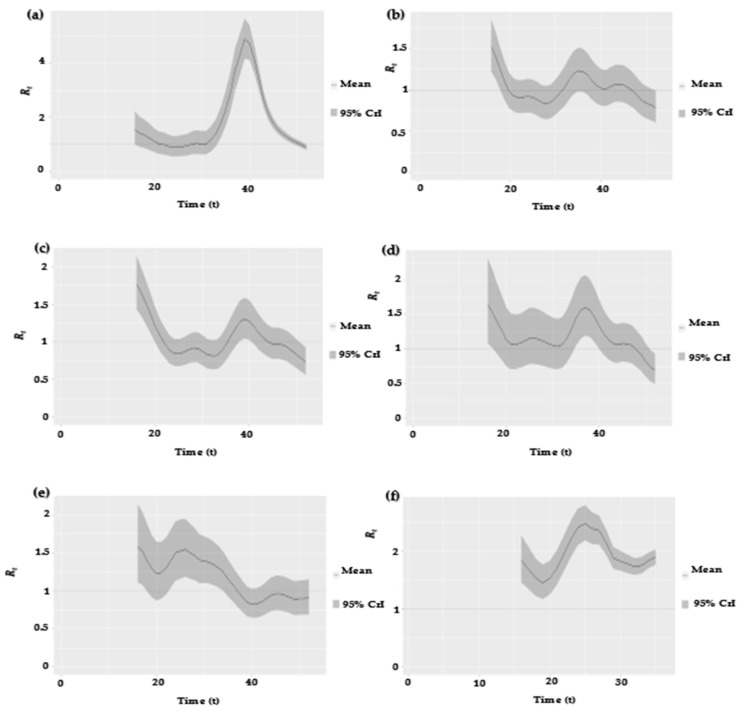
Estimated *R_eff_* series for the data from 1 January 2017 to 27 August 2022, represented by panels (**a**–**f**) using the serial interval from [25]. The solid line denotes the estimated mean, and the shaded gray area represents the 95% credible interval (CrI). The horizontal dashed line marks the level of *R_eff_* = 1. Note that $R_{t}$ = *R_eff_*.

Figure 2a–f shows the estimated $R_{t}$ based on the MPXV outbreaks data from 1 January 2017 to 27 August 2022. The estimated Rt reached an all-time peak in 2022, with a mean of 1.924 (95% CrI: 1.455, 2.485) and a median of 1.921 (95% CrI: 1.450, 2.482), since the last major epidemic in 2017, when the estimated $R_{t}$ had a mean of 1.8052 (95% CrI: 0.8817, 4.8732) and a median of 1.794 (95% CrI: 0.876, 4.863), using the SI from [25]. Similarly, the $R_{t}$ was estimated using the SI from Ward et al. [10], using different models (i.e., ICC and ICRTC); see Table 1 and Table 2 for summary values of the estimated $R_{t}$. Our $R_{t}$ estimates are consistent with the results obtained by [11]. In particular, in Table 1 and Table 2, we estimated the *R_t_* for the MPXV for the data from 1 January 2017 to 27 August 2022 to evaluate the instantaneous transmissibility of the MPXV outbreak. The result illustrates the mean and median of Rt for the MPXV transmission, which is helpful to public health policy planners in coming up with effective intervention strategies as well as in generating a sustainable policy to contain the outbreak in a timely manner.

## 4. Discussion

The transmission route of the MPXV in most of the recently affected non-endemic countries (via MSM) varies significantly from that in the endemic countries (which is mostly via human-to-human and zoonotic transmission from unidentified African rodents) [2]. The majority of the current MPXV scenarios in non-endemic countries are linked to cases imported from endemic countries. 

The MPXV and COVID-19 can be transmitted via contact with an infected person or contaminated materials. However, the transmissibility of COVID-19 appeared to be high [26]. While COVID-19 interventions—lockdown, social distancing, closure of gatherings—would have reduced transmissibility of infections, these measures were enforced late into the outbreak in many African settings. These could be some plausible explanations for the relative paucity of the MPXV cases in 2020 and 2021, with cases rising in 2022 when the COVID-19 measures had been dropped. As the control and the management of the COVID-19 pandemic rely hugely on a country’s healthcare system, most of the healthcare systems of the African countries have shown serious commitment. However, it is still not satisfactory with regard to containing the outbreak [21], which makes the countries more vulnerable to disease outbreaks. Thus, there is a need to constantly improve intervention measures to control the spread of diseases effectively.

To curb the MPXV epidemic in a timely and efficient manner whilst maintaining the COVID-19 control measures, we suggest providing adequate health resources and proper surveillance and contact tracing of the suspected, confirmed, and probable cases. This strategy is crucial in the preparedness plans against the possible severe outbreaks of the MPXV. Thus, there is a need to strengthen human and resource allocation to support the efforts to intensify the laboratory for proper diagnosis, surveillance, and response actions. This is in addition to the antiviral agent called tecovirimat, which was earlier developed for the smallpox virus and has been certified for use against the MPXV in 2022 (WHO, 2022a). The vaccine has been found to be up to about 85% effective in preventing the MPXV [2]. Therefore, public health organizations should collaborate fully with local governments to provide enough vaccines, especially in the most vulnerable communities/counties, to prevent more devastating scenarios that could occur due to the synergistic situation of the MPXV and the current pandemic of COVID-19.

We estimated *R_t_* for the MPXV outbreak in Nigeria for the data from January 2017 to August 2022. We observed that the *R_t_* reached an all-time peak in 2022 since the last major epidemic in 2017, which was plausibly due to the severe socioeconomic and public health consequences caused by the current COVID-19 pandemic [27]. Nevertheless, more data are needed to investigate the actual drivers of the current scenarios. The MPXV transmission increased significantly during the COVID-19 pandemic compared to two years before the pandemic, and the situation worsened to reach an all-time peak in 2022. This is in line with the current global situation, where the MPXV has been reported even in countries with no record of MPXV cases [2,5].

Nigeria and many other countries witnessed the unfortunate scenario of the overwhelmed healthcare system due to the synergistic impact of COVID-19 and other infectious diseases such as malaria and Lassa fever [28]. Thus, we suggest the urgent need to intensify awareness campaigns (even among healthcare workers) on the potential threats caused by the MPXV; this would help to reduce the risk of onward community transmission and improve preparedness plans. To this end, we plan to develop a more advanced epidemic model for MPXV transmission that will incorporate awareness programs and other helpful intervention measures to shed epidemiological insight and to disseminate information on the prevention and control of the MPXV transmission and, at the same time, sustain the COVID-19 control measures.

The socioeconomic deterioration, uncertainty, and complexity due to the pandemic’s impact, such as the collapse of small businesses, forcing people to abandon environmental hygiene and sanitization, with some even eating bushmeat more frequently in poor resource settings (which likely increased rodent-to-human interactions), could be potential drivers for the current MPXV upsurge [28,29,30,31]. Moreover, demography, particularly the population explosion in Nigeria from approximately 63 million in the 1960s to nearly 200 million people in 2022 in a non-enlarging Nigerian area, could be one of the critical factors that likely increases the animal–human interphase interactions and the evolving emerging zoonotic disease transmission risk [6]. However, more information is urgently needed to investigate the actual drivers of the current MPXV surge globally.

## 5. Conclusions

This study investigated the dynamics of the MPXV transmission in Nigeria. We discussed the probable variables that could have boosted rodent–human interactions, which are possibly due to the pandemic’s effects and other human behavioral or environmental factors. Most likely, these conditions facilitated the MPXV transmission in Nigeria and beyond. Most of the recent studies have forecasted that there will be an increase in MPXV cases worldwide before 2023 [8,10]. To produce more precise estimates, evaluate intervention measures, and enhance risk assessments globally, prospective epidemiological studies are urgently required. Based on the case notification in Nigeria, we estimated the $R_{t}$ of the MPXV outbreak and found that the Rt reached an all-time peak in 2022 with a mean of 1.924 (95% CrI: 1.455, 2.485) and a median of 1.921 (95% CrI: 1.450, 2.482). Real-time *R_t_* monitoring is practical and can give authorities vital data for situational comprehension and the adjustment of control tactics. Controlling the epidemic of the MPXV during the current COVID-19 pandemic requires substantial improvements in research, health resources, and awareness campaigns, especially in high-risk populations. These might boost reporting rates and avert onward community transmission. Additionally, a more precise estimation of the MPXV biological parameters such as Ro, exponential growth rate, and serial interval, will aid in understanding the dynamics of the virus and help in curtailing the transmission.

## Figures and Tables

**Table 1:** Summary results of the mean of *R_eff_* estimates for MPXV for the data from 1 January 2017 to 27 August 2022.

Year	Mean *R_eff_* (95% CrI) ^a^	Mean *R_eff_* (95% CI) ^b^	Mean *R_eff_* (95% CI) ^c^
2017	1.8052 (0.8817–4.8732)	1.4541 (0.9428–2.7914)	1.4793 (0.9492–2.8796)
2018	1.0204 (0.7854–1.5200)	1.0247 (0.8409–1.4185)	1.0279 (0.8374–1.4509)
2019	1.0385 (0.7316–1.7675)	1.0318 (0.7854–1.5785)	1.0360 (0.7809–1.6179)
2020	1.1760 (0.6978–1.6440)	1.1460 (0.7738–1.4712)	1.1544 (0.7699–1.5006)
2021	1.1693 (0.8257–1.5825)	1.1388 (0.8970–1.4670)	1.1472 (0.8969–1.4970)
2022	1.924 (1.455–2.485)	1.638 (1.383–1.868)	1.673 (1.410–1.908)

^a^ using serial interval from Guo et al. [25]; ^b^ using serial interval from ICC model from Ward et al. (2022); ^c^ using serial interval from ICRTC model from Ward et al. [10].

**Table 2:** Summary results of the median of *R_eff_* estimates for MPXV for the data from 1 January 2017 to 27 August 2022.

Year	Median *R_eff_* (95% CrI) ^a^	Median *R_eff_* (95% CI) ^b^	Median *R_eff_* (95% CI) ^c^
2017	1.794 (0.876–4.863)	1.4439 (0.9399–2.7858)	1.469 (0.942–2.874)
2018	1.0161 (0.7813–1.5144)	1.0204 (0.8365–1.4132)	1.0235 (0.8330–1.4456)
2019	1.0343 (0.7275–1.7613)	1.0277 (0.7811–1.5730)	1.0318 (0.7765–1.6123)
2020	1.1655 (0.6916–1.6237)	1.136 (0.767–1.453)	1.1441 (0.7632–1.4822)
2021	1.1627 (0.8218–1.5679)	1.1324 (0.8928–1.4534)	1.1408 (0.8920–1.4831)
2022	1.921 (1.450–2.482)	1.635 (1.378–1.866)	1.670 (1.405–1.905)

^a^ using serial interval from Guo et al. [25]; ^b^ using serial interval from ICC model from Ward et al. [10]; ^c^ using serial interval from ICRTC model from Ward et al. [10].

## Data Availability

All the data used are available in the public domain.
